# Biomechanical performance evaluation of S_2_AI combine with LC-2 screw for day II pelvic crescent fracture dislocation via finite element analysis

**DOI:** 10.1038/s41598-025-00156-6

**Published:** 2025-05-14

**Authors:** Xuan Pei, Jincheng Huang, Zhixun Fang, Shenglong Qian, Wei Zhou, Guodong Wang, Jianyin Lei, Ximing Liu

**Affiliations:** 1 Department of Orthopedics, General Hospital of Central Theater Command of PLA, Wuhan, 430070 Hubei Province China; 2 University Center of Orthopaedic, Trauma and Plastic Surgery, University Hospital Carl Gustav Carus at Technische Universität Dresden, 01307 Dresden, Germany; 3 Affiliated Second People’s Hospital, Three Gorges University, Yichang, 443000 Hubei Province China; 4 Department of Traditional Chinese Orthopedics and Traumatology, Xiamen Third Hospital, Xiamen, 361100 Fujian Province China; 5 Taiyuan University of Technology, Taiyuan, 030002 Shanxi Province China; 6 Hubei University of Chinese Medicine, Wuhan, 430065 Hubei Province China

**Keywords:** Trauma, Software, Computational models

## Abstract

Plate fixation is a classic method for treating day II crescent fracture dislocation of the pelvic (CFDP). However, due to the advantages of minimally invasive techniques and reduced complications associated with internal fixation percutaneous cannulated screws have emerged as a promising alternative for treating Day II CFDP. In this study, we propose using an S_2_AI screw combined with an LC-2 screw (S_2_AI + LC-2) for the treatment of Day II CFDP. The aim of this study was to compare its biomechanical stability with that of two conventional fixation methods using finite element analysis (FEA). A finite element (FE) model of pelvic was developed and validated. Three fixation methods were applied: S_1_ sacroiliac (SI) screws combined with LC-2 screw (S_1_ + LC-2), S_1_ and S_2_ SI screws combined with LC-2 screw (S_1_ + S_2_ + LC-2), and S_2_AI + LC-2. A 500 N load was applied, and the displacement of the crescent fracture fragments, the stress distribution of the implants, the displacement of the SI joint, and the maximum stress on the bone surrounding the screws were analyzed across the three FE models. After loading 500 N stress, the maximum displacement of the crescent fracture fragment and the maximum stress of bone around the implant in the S_2_AI + LC-2 group were the smallest in three groups. The displacement of SI joint in S_2_AI + LC-2 group was less than that in S_1_ + LC-2 and S_1_ + S_2_ + LC-2 (P < 0.001). The maximum stress of implants in each group is smaller than the yield stress of titanium. The maximum stress of the bone around the screws at SI joint in all models lower than the yield strength of cortical bone. The maximum stress of the bone around LC-2 screws in all models lower than the yield strength of cancellous bone. The S_2_AI + LC-2 group can achieve reliable stability of the SI joint, and the stress on the bone around the screw could be reduced. The S_2_AI + LC-2 group has good biomechanical stability and can be considered as a new implant to treat Day II CFDP.

Crescent fracture dislocation of the pelvic (CFDP) is a posterior fracture-dislocation of the sacroiliac (SI) joint, characterized by crescent fracture fragments. It is usually caused by high-energy trauma, such as traffic accidents, falls from heights, or crush injuries^1^. Day II CFDP involve between one-third and two-thirds of the SI joint, resulting in a medium-sized, stable crescent fracture fragment. Its incidence accounts for 46.6 ~ 47% of all CFDP cases^1,2^. For Day II CFDP, it is necessary to simultaneously fix the SI joint dislocation and crescent fracture fragment to restore the stability of the pelvic ring^1,3,4^. Historically, open reduction and internal fixation (ORIF) has been the standard approach for managing Day II CFDP^1–3^. However, this approach has notable drawbacks, including extensive soft tissue dissection, significant blood loss, and a higher risk of complications^1,3,4^. In recent years, an increasing number of researchers have adopted percutaneous cannulated screws, such as SI screws, LC-2 screws, and posterior iliac screws, as an alternative treatment for Day II CFDP. These techniques offer advantages such as minimal trauma, shorter operation times, reduced blood loss, and reliable fixation^5,6^.

In 2002, Starr et al. first proposed the use of LC-2 screws for treating pelvic crescent fractures, but they did not address SI joint fixation^7^. For Day II CFDP, LC-2 screws can be used to fix iliac wing fractures, while SI screws are primarily utilized for stabilizing SI joint dislocations^6^. Although SI screws can effectively stabilize the SI joint, it has certain limitations, including screw displacement or loosening, risks of neurovascular injury, and persistent postoperative pain, which may particularly affect outcomes in patients with osteoporosis or sacral deformity^8,9^.

In recent years, some scholars have begun to explore the use of S_2_-alar-iliac (S_2_AI) screws as a new implant for treating SI joint dislocation^10^. This screw provides reliable biomechanical stability due to its trajectory through three to four layers of cortical bone. Compared to SI screws, the S_2_AI screw is considered safer, as it avoids important neurovascular structures and other complex anatomical features in front of the sacrum. Additionally, S_2_AI screws can be inserted freehand or percutaneously, making them a viable option for stabilizing to the posterior pelvic ring injuries^11^. Given these advantages, we first proposed S_2_AI screw combined with LC-2 screw to treat Day II CFDP. We compared the biomechanical characteristics of the S_2_AI + LC-2 group with two conventional implants for treating Day II CFDP, aiming to provide a theoretical basis for further clinical application. Our hypothesis was that the S_2_AI + LC-2 group would offer sufficient stability and demonstrate the optimal performance in FEA.

## Materials and methods

### Establishment of day II CFDP model

**Fig. 1:**
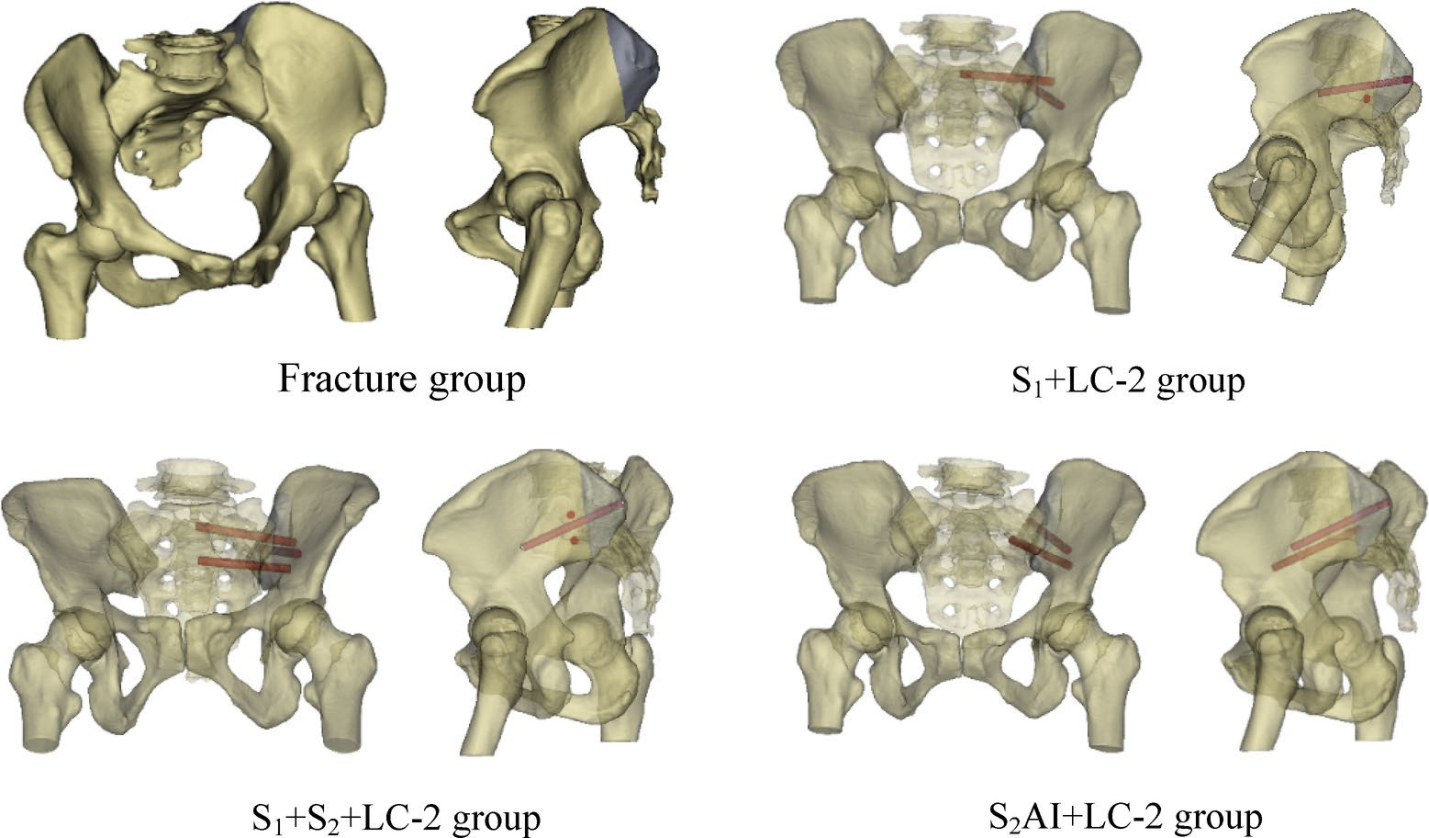
Four groups of fixation models in the study.

**Fig. 2:**
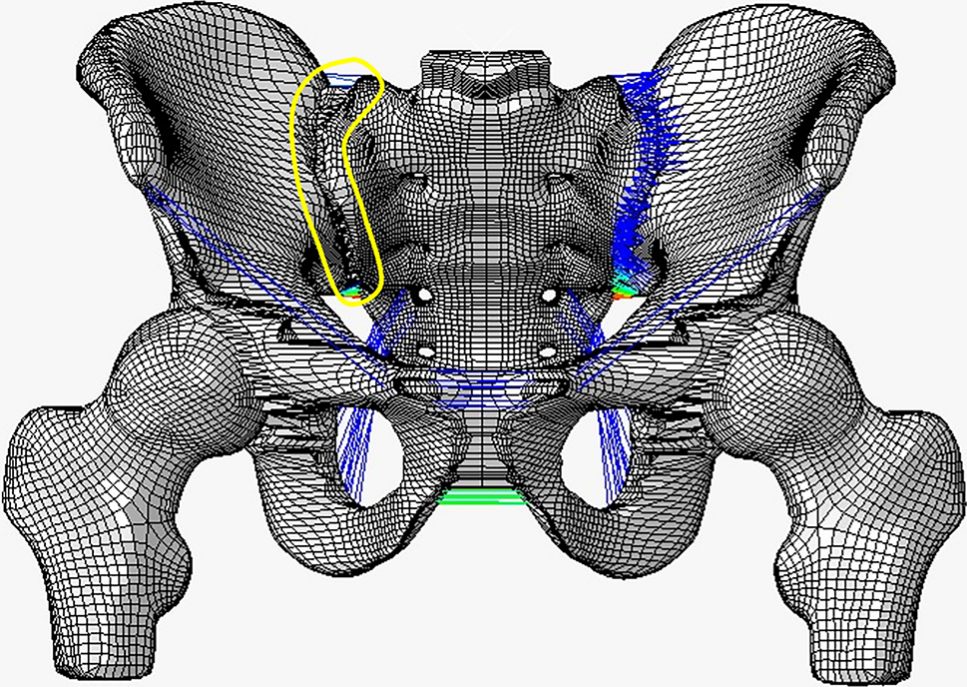
The SI ligaments in front of the iliac fracture line (the area outlined by a yellow border) were removed to simulate the SI joint dislocation.

Computed tomography (CT) images of a healthy 35-year-old female (165 cm, 55 kg, BMI 20.2) with no known pathologies were used to construct the FE model. The geometric model was built by importing saved DICOM format images into Mimics 21.0 (Materialize, Inc., Leuven, Belgium). Subsequently, the geometric model was refined using Geomagic Studio 10.0 (Geomagic Inc., USA). The geometrical model was then imported into Hypermesh 14.0 (Altair Inc., USA) to develop the bones, cartilages, and ligaments of FE model, which were divided into different tetrahedral mesh structures. The full pelvic includes the left ilium, right ilium, sacrum, symphysis pubis, and femoral bone, with the bones consisting of cortical bone and cancellous bone. According to the anatomical positions of key pelvic ligaments, the anterior SI, interosseous SI, posterior SI, sacrotuberous, sacrospinous ligaments, superior pubic and arcuate pubic ligament are created at corresponding nodes on the surface of the normal model. Relevant parameter settings are assigned based on previous literature reports^12^. The cortical bone of the sacrum and ilium were modeled as 1.5 mm thick shell. SolidWorks 2017 (Dassault Systèmes SolidWorks Inc., USA) was used to model the screw. The diameter of all screws is 6.5 mm, with screw lengths of 75 mm for the S_1_ and S_2_ SI screws, 80 mm for the LC-2 screws, and 75 mm for the S_2_AI screws. By combining the pelvic model and internal fixation, four groups of FE models were built (Fig. 1). The fracture line of Day II CFDP FE model starts from the S_2_ sacral foramen and then extends posteriorly and superiorly to terminate at the iliac wing (Fig. 1). The FE models were meshed into different tetrahedral mesh structures with Hypermesh14.0 (Altair Inc., USA). The nodes and element number of FE models are showed in Table 1. All implant materials were assumed to be titanium, with an elastic modulus of 110 GPa and a Poisson’s ratio of 0.3^13^ . The generated 3D model was imported into Abaqus 2020 software (SIMULIA, Inc., France) to define the bone contact surfaces, assign the corresponding material properties (Tables 2 and 3), and conduct FEA^14,15^. SI dislocation was simulated by removing the SI joint ligaments anterior to the iliac fracture line (Fig. 2).

**Table 1:** The nodes and elements of three groups of FE models.

FE model	Node number	Element number
Fracture group	210,272	113,419
S_1_ + LC-2 group	216,606	117,807
S_1_ + S_2_ + LC-2 group	220,113	120,239
S_2_AI + LC-2 group	217,006	118,087

**Table 2:** Material properties of pelvic model.

Component	Element type	Elastic modulus (MPa)	Poisson’s ratio
Cortical bone	C3D6	17,000	0.30
Cancellous bone	C3D6	150	0.20
Pubic symphysis	C3D6	5	0.45
Iliac endplate	C3D6	500	0.25
Sacroiliac joint cartilage	C3D6	25	0.30
Sacral endplate	C3D6	500	0.25
Sacral cartilage	C3D6	1000	0.30
Femoral cartilage	C3D6	1000	0.30

**Table 3:** Material parameters of the major pelvic ligament.

Ligament	Unit type	Stiffness (*N*/mm)
Inguinal ligament	T3D2	250
Sacroiliac ligaments	T3D2	5000
Sacrospinous ligaments	T3D2	1400
Sacrotuberous ligament	T3D2	1500
Superior pubic ligament	T3D2	500
Arcuate pubic ligament	T3D2	500

### Finite element model validation

According to the methods described in previous studies, the FE models were constructed^16^. The validity of our model was confirmed by comparing it with reported cadaveric and in vitro data^15,16^. In the vitro study, point loading was applied to the ventral and dorsal surfaces of the sacrum, located in the midsagittal plane of the lower S_1_ and upper S_2_ vertebrae. A node at the midpoint of the superior sacral endplate’s maximum diameter in the midsagittal and coronal planes was selected as the reference point for displacement measurements. FE model was validated with five translational loads (294 N) and three rotational moments (42 Nm) (anterior, posterior, superior, inferior, mediolateral, flexion, extension, and axial rotation).

### Loading and boundary condition

The distal ends of the femurs on both sides were constrained to simulate a standing posture. The six directions of the left and right ilium, the ischial tubercle surface, and the acetabulum were also constrained. In reference to previous studies, the middle of the top surface of S_1_was selected as the force loading location, and a vertical force of 500 N^18^ is applied. Binding constraints were applied among the ilium, sacrum, and SI joint, as well as between the screws and the surrounding bone surfaces, using Abaqus 2020 software. In addition, surface-to-surface contact were defined across the fracture surfaces.

### Data analysis

**Fig. 3:**
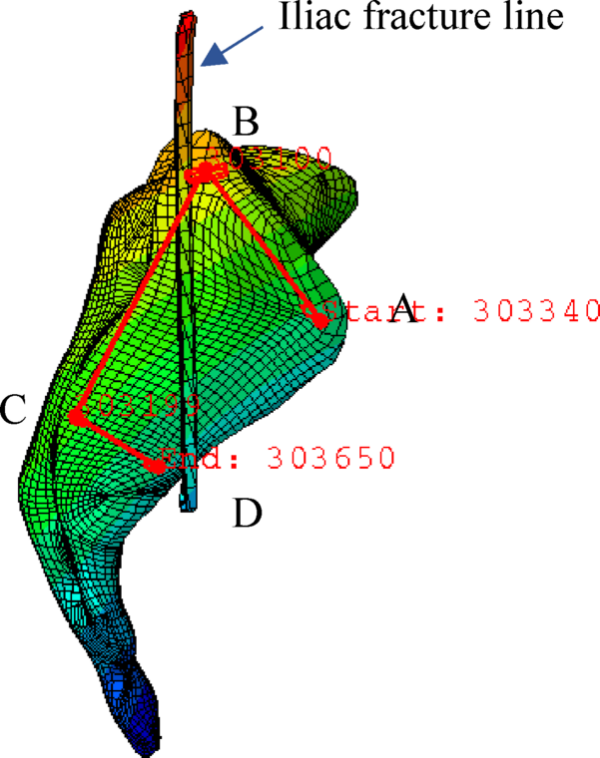
Four observation points of the SI joint. The anterior reference points (**A**,** B**) are indeed located in front of the iliac fracture line, where there is no SI joint ligament, while the posterior reference points (**C**,** D**) are located behind the iliac fracture line, where the SI joint ligaments are present.

The displacement of the crescent fracture fragment, the stress distribution of implants, SI joint displacement, and the stress on the bone around the screw were recorded and analyzed. The highest point of the front edge of right articular surface of sacrum (point A), the highest point of the posterior edge of right articular surface of sacrum (point B), the lowest point of the posterior edge of right articular surface of sacrum (point C), and the lowest point of the front edge of right articular surface of sacrum (point D) were selected as observation points for the SI joint (Fig. 3). The displacement value of each observation point was recorded and considered as the displacement of the SI joint.

## Results

### FE model validation

**Fig. 4:**
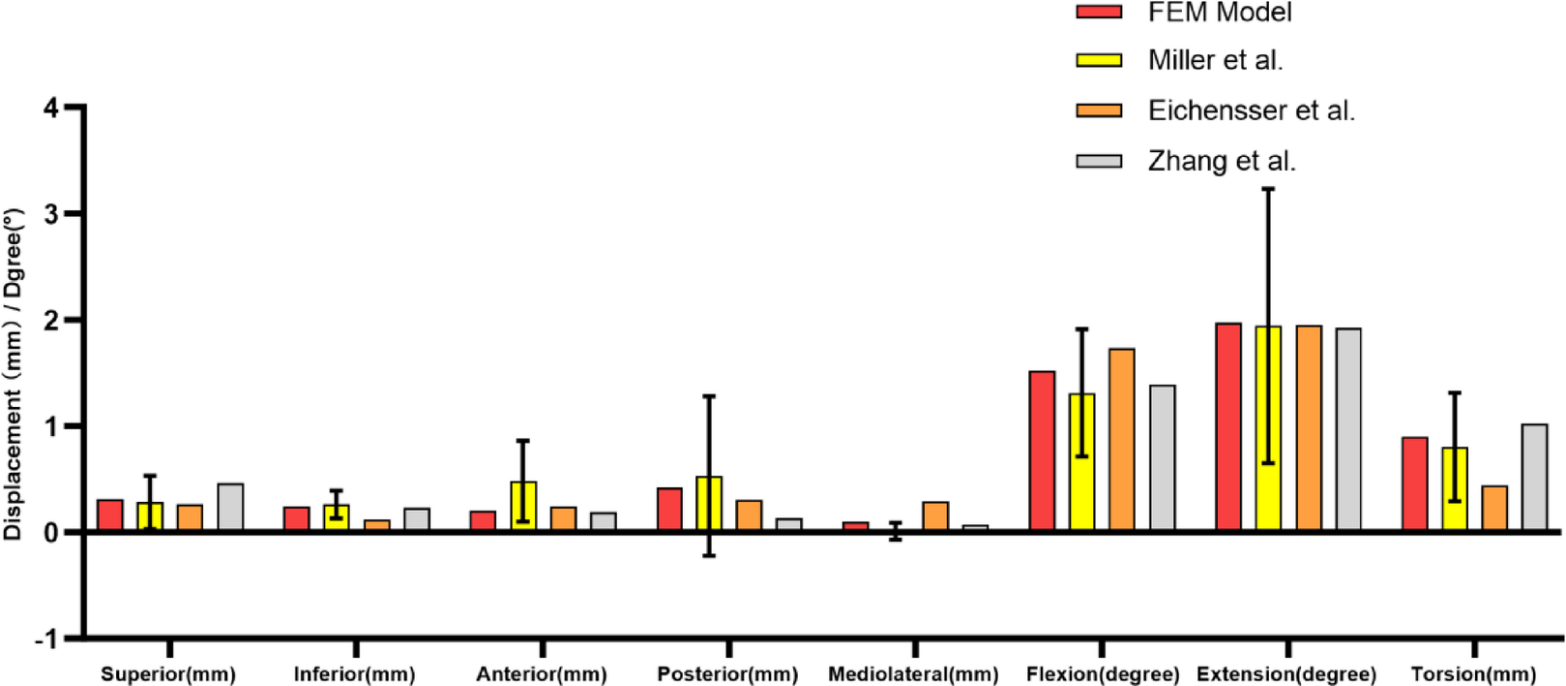
Comparison of sacral displacement between our finite element simulation and previous result.

This study is highly consistent with the previous research data reported by Eichenseer and Zhang, and the two groups of data and deformation trends are similar^15,16^. When compared with the results of the Miller model, it was found that all test data fell within the standard error range of the Miller model data, indicating good overall agreement^17^. The results were recorded and displayed in Fig. 4.

### Stability evaluation

**Fig. 5:**
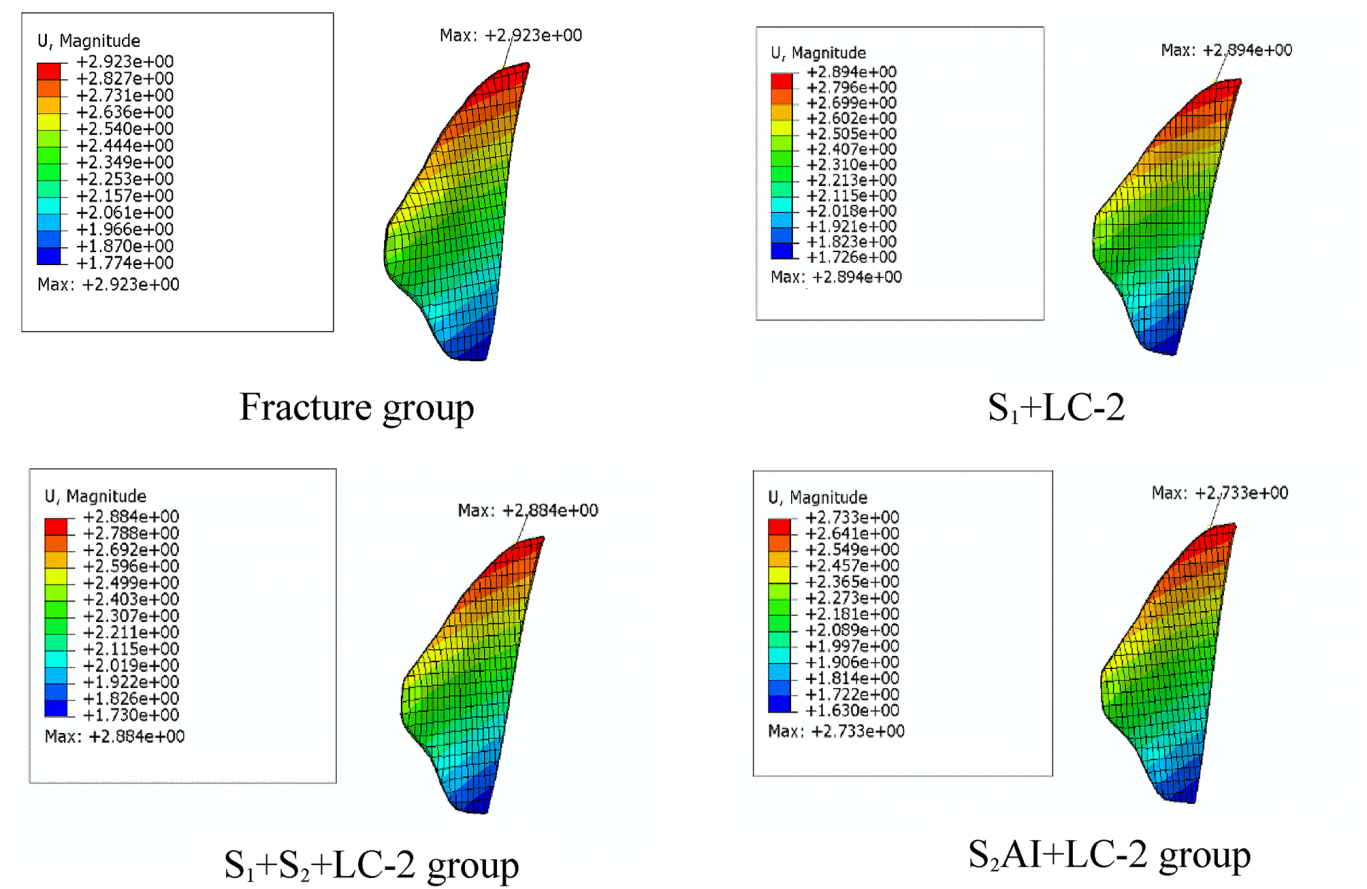
Displacement distribution nephogram of crescent fracture fragments in four groups.

**Fig. 6:**
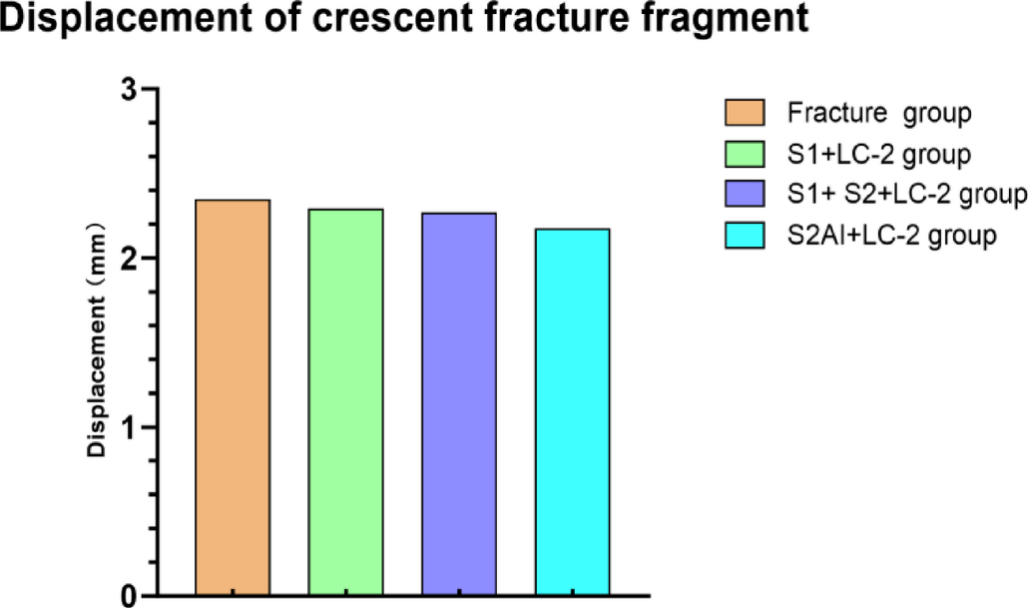
Displacement of crescent fracture fragment in four groups.

**Fig. 7:**
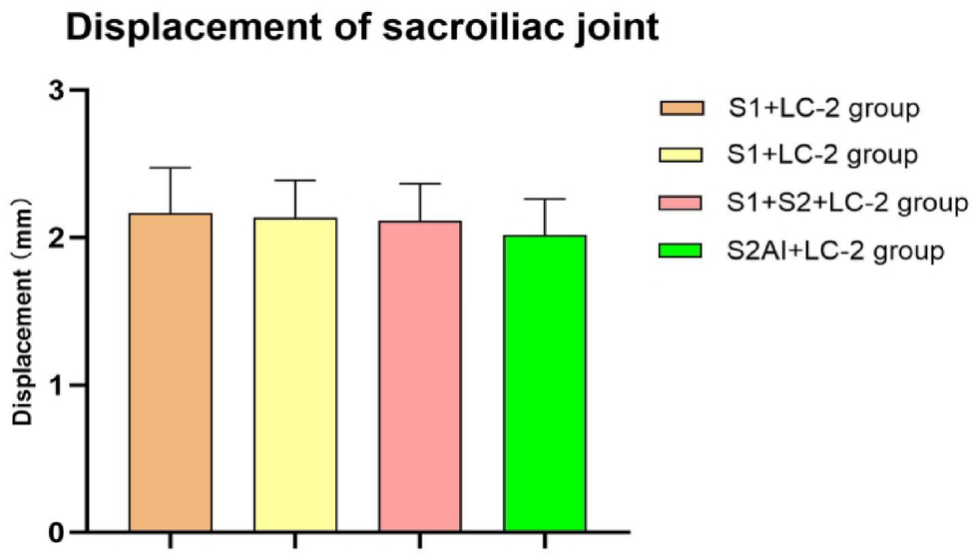
Displacement of SI joints in four groups.

The stability of internal fixation after implantation is evaluated by analyzing the following two indicators: (1) The displacement of the crescent fracture fragments (Figs. 5 and 6), and (2) The displacement of the SI joint (Fig. 7). The maximum displacements of the crescent fracture fragments are as follows: 2.923 mm in the Fracture group, 2.894 mm in the S_1_ + LC-2 group, 2.884 mm in the S_1_ + S_2_ + LC-2 group, and 2.733 mm in the S_2_AI + LC-2 group. The displacement values, in descending order, follow this pattern: Fracture group > S_1_ + LC-2 group > S_1_ + S_2_ + LC-2 group > S_2_AI + LC-2 group (Fig. 5). The displacement of the SI joint is 2.16 ± 0.30 mm in the Fracture group, 2.13 ± 0.25 mm in the S_1_ + LC-2 group, 2.11 ± 0.25 mm in the S_1_ + S_2_ + LC-2 group, and 2.01 ± 0.24 mm in the S_2_AI + LC-2 group. Similarly, these displacement values follow a descending order: Fracture group > S_1_ + LC-2 group > S_1_ + S_2_ + LC-2 group > S_2_AI + LC-2 group (Fig. 7). A smaller displacement value indicates a more stable implant.

### Maximum stress of implants

**Fig. 8:**
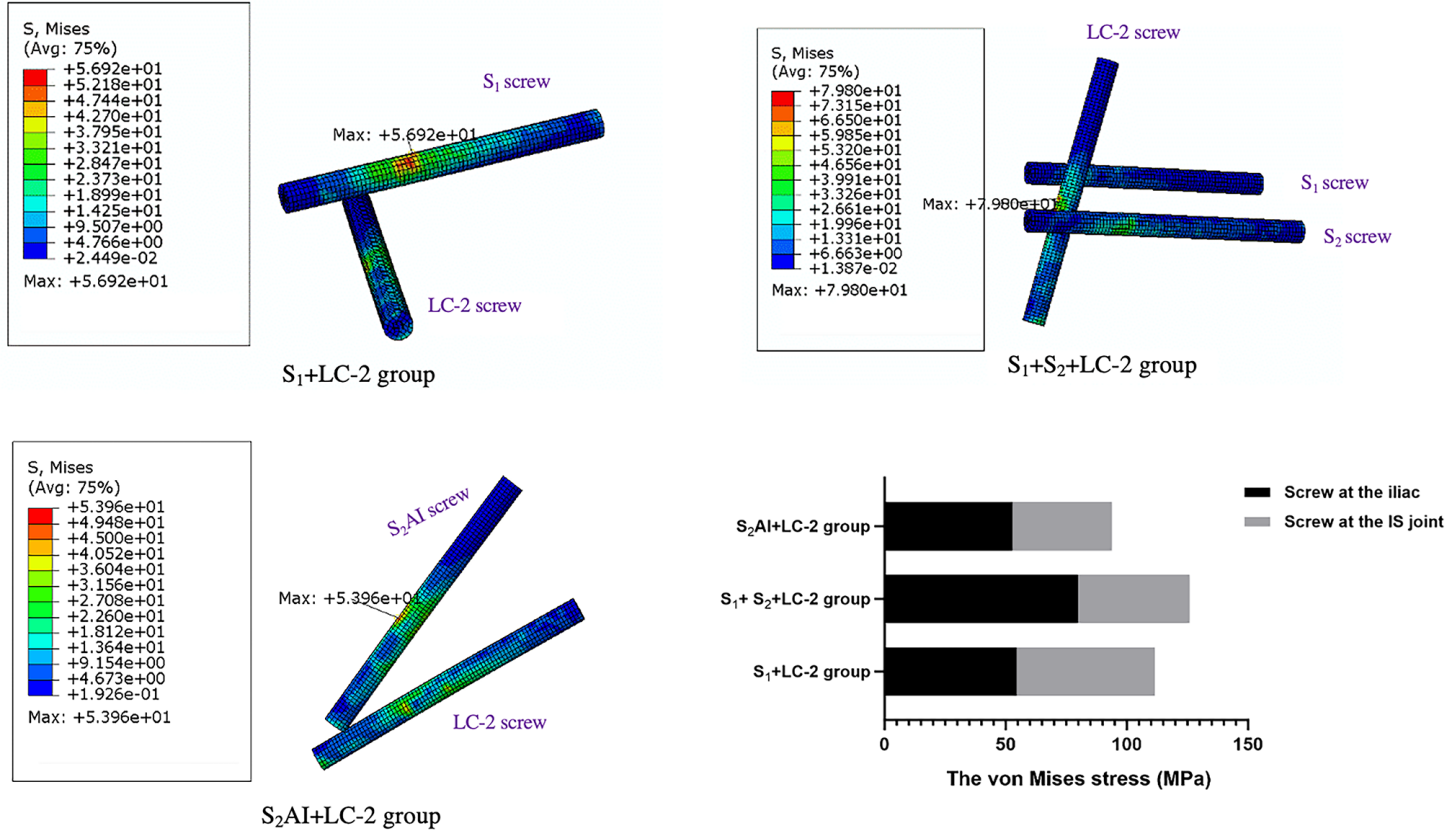
Stress nephogram of implants in the three groups.

The von Mises stress distribution of the three groups of implants is shown in Fig. 8. Differences in stress distribution on the implants were observed among the groups, with the S_2_AI + LC-2 group showing smaller variations. Among the three groups, the S_2_AI + LC-2 group exhibited the lowest von Mises stress on the SI joints and iliac. Additionally, the maximum von Mises stress of implants in all groups remained below the yield stress of titanium^19^.

### Bone stress distribution around implants

**Fig. 9:**
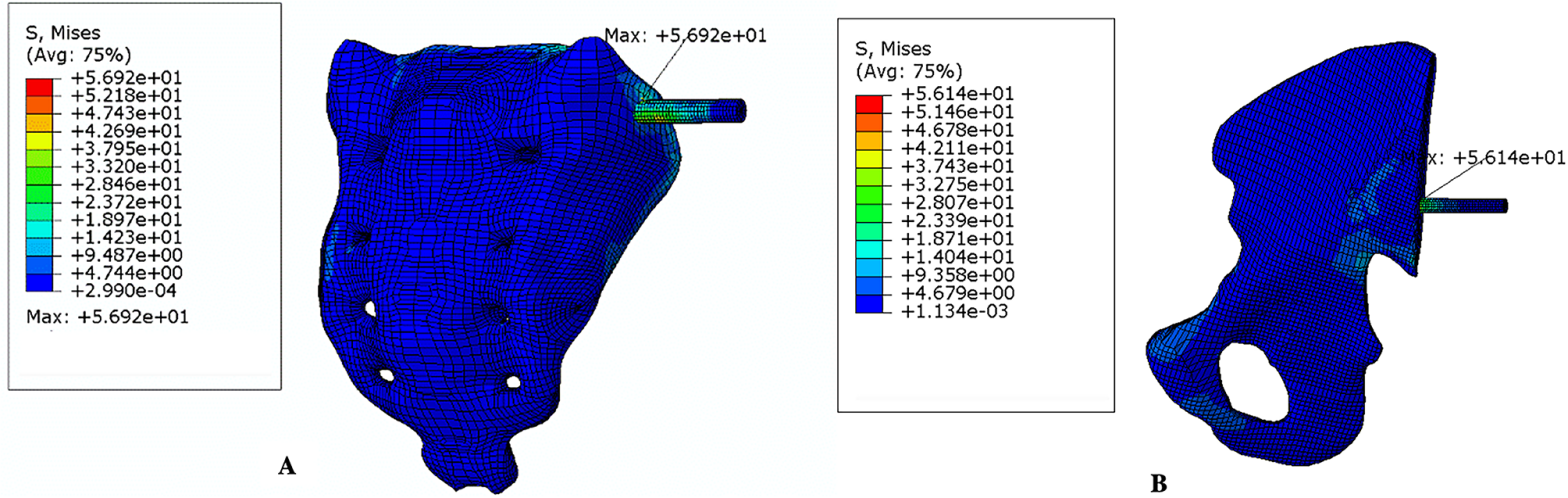
Nephogram of maximum stress concentration points in implant. (**A**) Maximum stress concentration point of screws at SI joint. (**B**) The maximum stress concentration point of LC-2 screws at iliac.

According to the nephogram of implants, the stress peak of implants at the SI joint is concentrated on the contact surface between the implant and the cortical bone of the sacrum. For the LC-2 screw, the maximum stress peak is concentrated on the cancellous bone on the iliac side (Fig. 9). Among the three groups, the S_2_AI + LC-2 group showed the lowest bone stress around the implants (Table 4), thereby reducing the risk of screw loosening. In all models, the maximum stress on the bone around the screws at the SI joint was lower than the yield strength of cortical bone^20^. Similarly, the maximum stress on the bone surrounding the LC-2 screw in all models was below the yield strength of cancellous bone.

**Table 4:** Displacement of the SI joint and the maximum von Mises stress on the bone around the screws.

Group	Displacement of SI joint observation points (mm)				Mean ± SD	The maximum von Mises stress in the bone around the screw (MPa)	
	A	B	C	D		Iliac screws	SI joint screws
Fracture group	1.935	2.587	2.196	1.945	2.16 ± 0.30	-	-
S_1_ + LC-2 group	1.962	2.479	2.168	1.920	2.13 ± 0.25	17.08	16.120
S_1_ + S_2_ + LC-2 group	1.942	2.457	2.146	1.898	2.11 ± 0.25	13.50	0.430
S_2_AI + LC-2 group	1.853	2.347	2.051	1.815	2.01 ± 0.24	0.245	0.215

## Discussion

In recent years, scholars have begun to use percutaneous cannulated screw treatment for Day II CFDP. Common strategies include: SI screw combined with posterior iliac screw or LC-2 screw fixation^5,4^, simple SI screw fixation^21^. Recently, Zhang et al. found that S_2_AI screws can also be used to treat SI dislocation with sufficient mechanical stability^10^. Based on these findings, we propose a novel fixation strategy combining S_2_AI screw with LC-2 screw for the treatment of Day II CFDP. In this study, we investigated the mechanical differences in the treatment of Day II CFDP using the following approaches: S_1_ SI screw + LC-2 screw (S_1_ + LC-2 group), S_1_ + S_2_ SI screws combined with LC-2 screw (S_1_ + S_2_ + LC-2 group), and S_2_AI screw + LC-2 screw (S_2_AI + LC-2 group). This computational analysis allowed us to uncover several interesting findings: (1) The S_2_AI + LC-2 group exhibited the least displacement of the crescent fracture fragment and the SI joint, and provided the best biomechanical stability among the three groups. (2) The stress difference in the screws at the iliac and SI joint was smaller between the S_1_ + LC-2 and S_2_AI + LC-2 groups than in the S_1_ + S_2_ + LC-2 group. (3) Among the three groups, the bone stress around the implants in the S_2_AI + LC-2 group was the smallest, which reduced the risk of screw loosening. (4) Differences were observed in the location of bone stress concentration around the implants. The maximum peak of stress on the implant at the SI joint was concentrated on the contact surface between the screw and the cortical bone, while the maximum stress of the LC-2 screw was concentrated on the cancellous bone.

In order to obtain reliable study results, it is essential to accurately construct the FE model and verify its validity. Day II CFDP involves a crescent fracture fragment of the iliac accompanied by SI joint dislocation^1^. In early FE studies, SI joint dislocation was simulated by removing the anterior ligament of the SI joint^22^. However, due to the absence of detailed descriptions regarding the location and orientation of the iliac fracture line, the specific site of ligament removal could not be clearly defined. As a result, the extent of ligament removal was often inconsistent, leading to either excessive or insufficient modeling of the dislocation. Furthermore, the lack of model validation in some previous FE studies has raised concerns regarding the reliability of their simulation results. To ensure the accuracy of our analysis, we validated our pelvic FE model by following the methodology described by Zhang et al. and Miller et al.^15,17^. The validation results indicate that our model closely approximates the anatomy and biomechanical behavior of a normal human pelvis, thereby enhancing the credibility of the subsequent analysis.

Titanium screws can withstand a maximum stress of 795 MPa^23^. Among the three implant groups, the maximum von Mises stress values in all implants were below the yield strength of titanium, indicating no risk of screw breakage under the simulated loading conditions. According to the principle of force interaction, when a force is applied to the pelvis, the screws transmit force on the surrounding bone. If the von Mises stress in the bone around the screws becomes excessive, it may lead to local bone damage or even fracture. Therefore, in addition to evaluating implant strength, it is equally important to assess the stress distribution in the surrounding bone. Theoretically, the yield stress of cancellous bone ranges from 5.8 to 10.8 MPa, while the yield stress of cortical bone is approximately 50 times that of cancellous bone^20^. In the present study, the stress nephogram of the pelvis in the three groups showed that the maximum stress on the bone around the screws at the SI joint in all models was lower than the yield strength of cortical bone. Similarly, the maximum von Mises stress on the bone around the LC-2 screw remained below the yield strength of cancellous bone across all models. Furthermore, the maximum von Mises stress on the bone surrounding the screws in the S_2_AI + LC-2 group was lower than that in the S_1_ + LC-2 group and the S_1_ + S_2_ + LC-2 group. This indicates that the S_2_AI + LC-2 group can effectively reduce stress on the bone around the screws, thereby lowering the risk of postoperative fractures and offering the highest safety among the three groups. Since the risk of screw loosening is primarily influenced by the magnitude of stress at the screw–bone interface, a lower stress level in the surrounding bone corresponds to a reduced likelihood of loosening. Therefore, these findings also indicate that the S_2_AI + LC-2 group may minimize the risk of screw loosening, further supporting its biomechanical advantage.

According to literature reports, displacement in FE models is commonly used to evaluate biomechanical stability^24,25^. In our study, we found that the displacement of crescent fracture fragments and the SI joint was highest in the S_1_ + LC-2 group, lowest in the S_2_AI + LC-2 group, and intermediate in the S_1_ + S_2_ + LC-2 group (Table 4). Previous studies, including the study by Cai et al., have demonstrated that the S_1_+ LC-2 group provides sufficient biomechanical stability through biomechanical testing^23^. Our findings further confirm this, but also reveal that the S_2_AI + LC-2 group exhibits even greater stability. This biomechanical advantages are particularly important for patients with severe SI joint instability or those with osteoporosis, as this fixation method offers greater resistance to displacement and better load-bearing capacity. It is worth noting that Day II CFDP can be accompanied by sacral fractures, which can further compromise the stability of the SI joint. In such cases, the addition of S_2_ SI screws is recommended to further enhance SI joint stability. In this study, we used one screw to fix the crescent fracture fragments. As is showed in (Figs. 4 and 5), our results indicate that the stability of the crescent fracture improves as SI joint stability increases. This can be attributed to the anatomical connection between the crescent fragment of the iliac wing and the sacrum, which is maintained via the intact portion of the posterior ligament complex.

This study has some limitations: (1) The results of FEA research are only calculated and analyzed by computer simulation, and further mechanical experiments on cadaver specimens are needed. (2) In this study, we only modeled the major ligaments, but in practice other ligaments and muscles may equally play an important role. Therefore, there may be differences between our simulated data and actual cadaver specimen model data. (3) In addition, this study only simulated non-osteoporotic crescent fractures and did not consider the impact of osteoporosis on internal fixation, which needs further study in subsequent experiments. (4) The thread is not considered in the FE model, which may have a certain potential impact on the bone stress around the screw, which needs to be further studied in subsequent experiments.

## Conclusion

In summary, the three groups of implants can achieve reliable biomechanical stability for the treatment of Day II CFDP, and the possibility of implant failure is unlikely. The results confirm that S_2_AI + LC-2 group could not only achieve reliable stability of the SI joint but also effectively reduce the stress of the bone around the screws and, to a certain extent, reduce the incidence of implant failure and screw loosening.

## Data Availability

The datasets used and/or analysed during the current study available from the corresponding author on reason- able request.
